# Method for Solving Difficulties in Rhythm Classification Caused by Few Samples and Similar Characteristics in Electrocardiograms

**DOI:** 10.3390/bioengineering10020196

**Published:** 2023-02-02

**Authors:** Jaewon Lee, Miyoung Shin

**Affiliations:** 1 Bio-Intelligence & Data Mining Laboratory, School of Electronic and Electrical Engineering, Kyungpook National University, Daegu 41566, Republic of Korea; 2 School of Electronic and Electrical Engineering, Kyungpook National University, Daegu 41566, Republic of Korea

**Keywords:** ECG rhythms, rhythm classification, deep learning, convolutional neural network (CNN)

## Abstract

A method for accurately analyzing electrocardiograms (ECGs), which are obtained from electrical signals generated by cardiac activity, is essential in heart disease diagnosis. However, rhythms are typically obtained with relatively few data samples and similar characteristics, making them difficult to classify. To solve these issues, we proposed a novel method that distinguishes a given ECG rhythm using a beat score map (BSM) image. Through the proposed method, the associations between beats and previously used features, such as the R–R interval, were considered. Rhythm classification was implemented by training a convolutional neural network model and using transfer learning with the created BSM image. As a result, the proposed method for ECG rhythms with small data samples showed significant results. It also showed good performance in differentiating atrial fibrillation (AFIB) and atrial flutter (AFL) rhythms, which are difficult to distinguish due to their similar characteristics. The performance for rhythms with a small number of samples of the proposed method is 20% better than an existing method. In addition, the performance based on the F-1 score for classifying AFIB and AFL of the proposed method is 30% better than the existing method. This study solved the previous limitations caused by small sample numbers and similar rhythms.

## 1. Introduction

An electrocardiogram (ECG) is a recording of the electrical response of a heart caused by its movement; thus, an ECG can be used to determine the activity of the heart [1]. It is generally used to identify a patient’s health condition by classifying the status of each stage of cardiac activity through changes in the ECG signals’ form or value; hence, these signals can be the most intuitive data in determining heart disease. The task of analyzing an ECG and classifying a patient’s abnormal symptoms based on the ECG characteristics is very important in determining the health of the patient’s heart. A typical disease studied using ECG is arrhythmia, which refers to an irregular pattern or phenomenon occurring in an ECG. It is classified according to the type of occurrence: atrial fibrillation (AFIB), atrial flutter (AFL), and sinus bradycardia (SBR), which are rhythmic conditions that have been identified as major threats to a heart’s health [2,3].

Experts visually check the waveforms of ECG signals and classify them based on their experience and background knowledge of cardiac rhythms [4]. However, due to the many patterns of heart activity and various environmental factors, diagnosis based on experience and background knowledge can lead to misjudgments, which hinders the timely application of an appropriate treatment method [5]. Indeed, the number of patients suffering from AFIB has been increasing, and about 15% of deaths caused by heart disease worldwide are caused by such misjudgments [6,7,8]. Therefore, if ECG signals are analyzed well, and cardiac rhythms are identified more accurately, an accurate diagnosis is likely possible.

In the past, ECG signals were analyzed by methods based on experts’ knowledge and finding known abnormal patterns. For example, ECG features (e.g., time elapsing between two consecutive R waves in an ECG, called the R–R interval) were calculated and judged by these methods. Meanwhile, a machine learning technique has been used to classify heart-related abnormal symptoms through learning based on collected data, which were accumulated gradually from patients [9,10]. However, previous studies have focused mainly on finding locations that correspond to R peaks in ECG signal patterns and classifying the types of beats found [11,12,13].

Recently, methods using deep learning have been used in various fields and problems, and such methods have been used in analyzing ECG signals (e.g., classifying AFIB and a rhythm) [14,15,16,17,18,19,20,21,22]. Nevertheless, a problem in analyzing an ECG is the difference in the number of data samples. Depending on the frequency of occurrence of rhythms, there could be few and many samples for rhythms with low and frequent occurrence, respectively. For this reason, an imbalance among rhythms exists in ECG databases such as the Massachusetts Institute Of Technology-Beth Israel Hospital (MIT-BIH) arrhythmia database, which is widely used and public [23]. This imbalance is a hindrance when learning and distinguishing various ECG rhythms, and it causes difficulties during the classification of correct ECG rhythms. For these reasons, research on how to classify multiple rhythms is insufficient. However, as mentioned above, the limitations are the difficulty of classifying rhythms using relatively few samples from the measured data and properly distinguishing similar patterns or characteristics between rhythms [24,25,26,27].

In this paper, to compensate for these limitations, we maximized the information present in an ECG. In analyzing rhythms, we assumed that the arrangement of beats in ECG signals is correlated with ECG rhythms. We then used the arrangement pattern of ECG beats to differentiate the various rhythms. In addition, we developed a method that considers not only these patterns but also features such as the R–R interval related to rhythm classification.

Through the proposed method, we tried to solve the difficulties caused by rhythms with similar characteristics and the existing limitations of imbalanced data. To achieve these goals, two datasets were constructed to classify various rhythm labels by the proposed method using MIT-BIH arrhythmia data. Finally, we determined the performance of the proposed model. Our main contributions can be summarized as follows.

1. We consider ECG rhythm as an arrangement of beats and classify various ECG rhythms by utilizing the arrangement of this pattern of beats. For this purpose, a series of ECG beat segments divided by a specific criterion are obtained from each ECG rhythm and are employed to train a beat classification model. The prediction score vector for each beat segment in the classification model is then used to generate an arrangement pattern of beats for each ECG rhythm. In doing so, some changes in ECG rhythms of the same or different types can be reflected as much as possible through the prediction score vector of the beat classification model.
2. The arrangement pattern of beats for a given ECG signal is converted into a beat score map (BSM) image, of which a continuous wavelet transform (CWT) is then fed to a deep convolutional neural network (CNN) for rhythm classification. Unlike existing methods that mostly focus on the overall characteristics of various ECG rhythms in the time or frequency domain, we subdivide the rhythms into a series of beat segments and characterize each beat segment by the prediction score vector of the beat classification model. The prediction score vectors for a series of beat segments are aligned along with time interval padding, leading to the production of the BSM image.
3. The proposed method is effective in classifying various types of ECG rhythms with data imbalance problems. Particularly, certain types of ECG rhythms with few samples can be distinguished well from other types with many samples. In addition, our method can well distinguish different ECG rhythms of similar characteristics, such as between AFIB and AFL, which have been known to be difficult to classify in previous studies.

The remainder of this paper is organized as follows: Section 2 presents existing recent research related to ECG rhythms. Section 3 describes the overall details of the proposed method such as BSM image generation and structure of network. Experiments and results are shown in Section 4 and Section 5, respectively. Conclusions are provided with some discussion in Section 6. 

## 2. Related Works

Hand-crafted features based on background knowledge have been extracted and used to analyze ECG signals. Recently, feature extraction, which is not done by humans, was conducted for the desired purpose of using deep learning [22,28]. Through these studies, hand-crafted features based on existing background knowledge were obtained by using machine learning [29,30,31]. In deep learning, the trend is toward automatically obtaining the most suitable features by methods such as CNN [32,33,34], long-short term network (LSTM) [11,35,36], and CNN autoencoder [37]. In a recent study [38], matrix images using the correlation between multi-channel EEG signals were created and analyzed using a deep CNN. As such, methods of imaging various physiological signals and analyzing them through CNN have been proposed.

Research has been undertaken on classifying ECG beats included in an ECG signal. In previous studies, QRS complexes that are features created based on knowledge of repetitive morphological patterns of ECG signals have been used [39]. However, rather than quantifying and analyzing the repeated patterns in a beat classification, studies have suggested the learning of the ECG signal’s one-dimensional (1-D) data itself to suit beat classification [40,41,42]. Alternatively, the ECG signal is converted to an image through transformation for use as an input of a 2D deep learning method [3,43]. Notably, the method of converting and utilizing ECG using CWT to compensate for possible limitations in an existing Fourier transform is performing well [44,45].

In addition to ECG beat classification, studies have been conducted to identify the rhythm of ECG signals [28]. Among these studies, the most active is in the field of single rhythm classification using AFIB. AFIB is a common ECG rhythm in many databases; thus, it is suitable for research. Methods for AFIB classification calculate features related to AFIB, and they analyze changes in the calculated features [46,47,48]. Current methods have preferred using deep learning over traditional features. Recent studies have integrated the use of CNNs and other network structures, and they obtained good performance for classifying ECG rhythms [49,50,51,52,53]. In addition, research is being conducted on real-life applications to reduce computational costs while maintaining the performance of deep CNNs. [54]. However, they have not paid much attention to the various ECG rhythms existing in real life. As such, studies on single rhythm classification have shown good performance but failed to distinguish well between several rhythms.

Meanwhile, many studies have been conducted to classify several rhythms [26,27,55,56,57]. However, they have shown severe degradation in classifying some rhythms with data imbalance problems. Moreover, they had some difficulties in differentiating between ECG rhythms of similar characteristics, such as between AFIB and AFL.

Finally, many researchers have studied the beat and rhythm of ECG signals using various features and network structures [58,59,60]; however, we have not found research on the correlation between the listings of beats and rhythms. Thus, we intend to solve the problem of the existing threshold by utilizing the association between the listings of beats and rhythm in the proposed method.

## 3. Methods

The proposed model for classifying ECG rhythms employs a 10-s ECG signal as an input. The proposed method is divided into three main parts: (1) beat unit analysis that trains a beat classification model using CWT and obtains a beat score vector from the trained model; (2) the creation of a BSM image by integrating the obtained beat score vector into a beat score matrix and applying time interval padding based on the R-peak position in the matrix; and (3) rhythm classification implemented by utilizing the information related to the existing feature and the correlation between the list of beats in an ECG signal and ECG rhythms.

**Figure 1:**
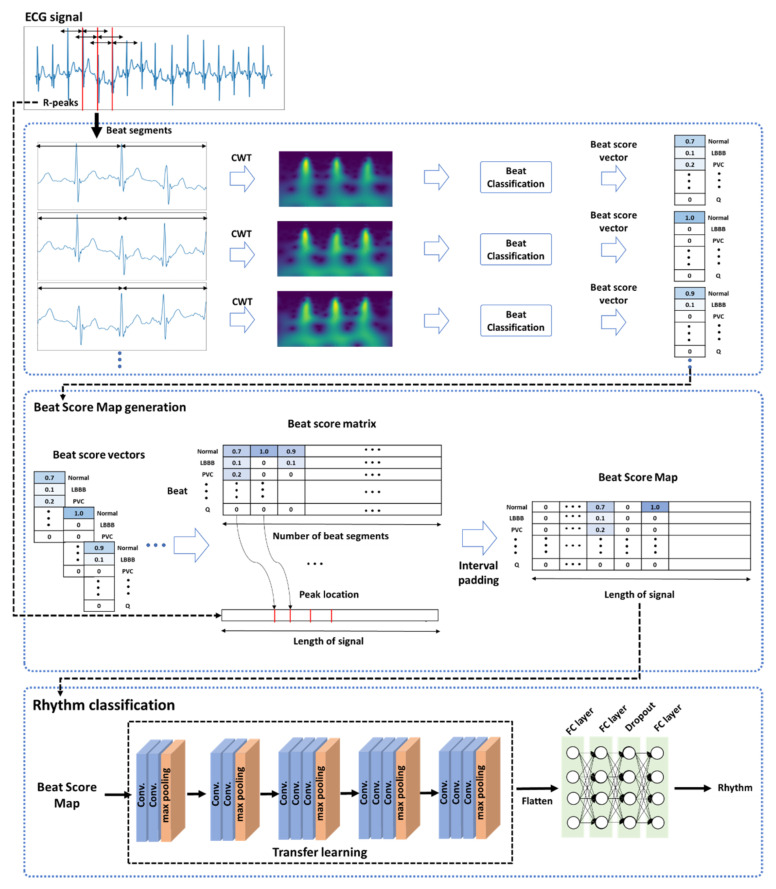
General overview of the proposed method.

A general overview of the proposed model is provided in Figure 1. In the first part, a given ECG signal of an input is created with beat segments based on R peak detection, and the ECG signal of the created segments is then converted into an image by the CWT method. The converted image is applied to classify the beats contained in a segment. As a result, we construct a beat score vector by taking the predicted score values rather than the label values for each beat class to preserve information about differences in ECG signals. In the second part, the beat score vectors created from each segment are merged with interval padding to form a beat score matrix, where the interval padding is applied to consider the R–R interval in the created matrix. For this purpose, the location of the R peaks used to create the beat segments is utilized. Thus, beat score map (BSM) images conveying both listing patterns of beats and R–R intervals are created by this part of the method. In the third part, a two-dimensional (2-D) convolutional neural network (CNN) structure model is trained using the created BSM images by transfer learning, and the ECG rhythms are classified.

### 3.1. Preprocessing

We carry out the removal of baseline drift and high-frequency noise in a signal by discrete wavelet transform. For removing baseline drift, the decomposition scale was set to 9 in Daubichies-4 (db4). For high-frequency noise filtering, the decomposition scale was set to 6 in the same db-4, and the frequencies ranging from 50 Hz to 100 kHz were filtered. In rhythm classification, each window was slid every 1 s, and the rhythm label corresponding to a sequence in each 10-s unit was recorded.

### 3.2. BSM Image Generation

To compensate for known limitations, BSM image generation is designed to utilize beat prediction scores obtained in the beat classification process and information from the R–R interval through interval padding.

#### 3.2.1. Beat Classification

Beat classification was implemented based on the structure of a recently studied method that creates a spectrogram image using ECG signal CWT [44]. To create images by CWT, a 2.4-s chunk for each R-peak position was created, and each chunk consisted of ECG signals from 1.2 s before to 1.2 s after an R peak. Using the created images as input, the model was trained via images created for the classification of the given beat.

#### 3.2.2. Interval Padding and Resizing

A beat score vector for each beat was obtained using the prediction score value from the learned beat classification model. All vectors included in a 10-s ECG segment were merged to form a beat score matrix. If the vectors contained in an ECG signal were combined simply to form a matrix, the association between the beats and rhythms was considered; however, information on the R–R interval associated with the rhythm classification was lost. Thus, a BSM image was created by applying interval padding in which the beat score vector was entered at the location where the R peak exists, while the rest were filled with zero. Information such as the location of the overall R peaks within a unit ECG signal and the R–R interval obtained therefrom were also included in the BSM image.

**Figure 2:**
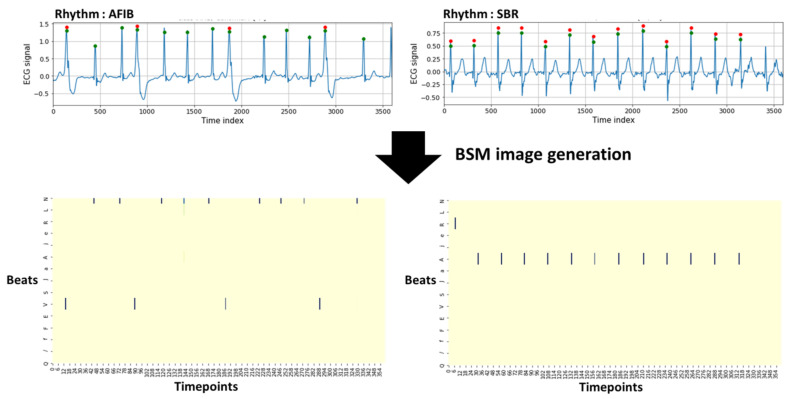
BSM image generation of an ECG signal.

BSM images were produced initially in a 3600 × 15 structure because the data used in the experiment had a sampling rate of 360 Hz, resulting in 3600 time points in 10 s. The size was reduced by one-tenth and adjusted to 360. In a value corresponding to 15, the number of labels for all beats in the provided data is given. The created matrices resized to 360 × 150 are suitable for CNN learning. The structure of a created 360 × 15 matrix is shown in Figure 2. The x- and y-axis represent the 360 time points and each beat label, respectively. The figure confirms that the beat interval, beat type, and prediction score value for each beat are configured differently for each rhythm class.

### 3.3. BSM Image Classification

Based on the created BSM image, rhythm classification was implemented by a 2D CNN. For image classification by transfer learning, the commonly used 2D CNN structure of VGG16 [61] was utilized. Therefore, the pre-trained weight was the initial value, and the weight was learned newly with a given image. The network consisted of five convolution blocks and three layers that were connected fully in a large structure.

## 4. Experiments

The proposed method used labels for the rhythm of ECG records obtained from the MIT-BIH arrhythmia database and the locations of R peaks. We constructed two ECG rhythm datasets using the given rhythm labels, and the performance of the method was evaluated for each dataset.

### 4.1. Dataset

**Figure 3:**
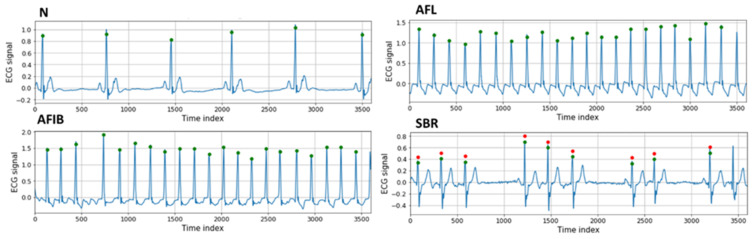
Representative ECG rhythm sequences.

In our experiment, we used the MIT-BIH arrhythmia database containing 48 half-hour records with various labels. The database was measured in MLII and V5 at a sampling rate of 360 Hz from two leads. The experiment was conducted using only the MLII among the two leads. Annotations created by two or more experts are provided for each record, and they provide the locations of beats based on the R peak. The labels of the beats in 16 categories are also provided, which also provide the labels for ECG rhythms within each record. Sections corresponding to each rhythm in the database were defined as the label of the rhythm. The beat label ‘?’ was excluded because it was not found; thus, only 15 beat labels were used in the experiment. The rhythm labels were the following: normal sinus rhythm (N), AFIB, AFL, SBR, supraventricular tachyarrhythmia (SVTA), ventricular bigeminy (B), ventricular trigeminy (T), and paced rhythms (P). Representative 10-s ECG segments for four rhythm labels, N, AFL, AFIB, and SBR, are presented in Figure 3. The green and red circles represent the R-peak locations and beats (not normal beats), respectively.

We configured two ECG rhythm sets for various ECG rhythms. First, we classified five rhythm classes: N, AFIB, SVTA, B, and T, which aimed to evaluate the performance of the model for rhythms with relatively few samples. Then, we evaluated AFIB and AFL, which are difficult to classify owing to their similar ECG rhythms. The second dataset consisted of six ECG rhythm classes: N, AFIB, B, P, AFL, and SBR. The number of samples per rhythm in each experimental dataset is summarized in Table 1 and Table 2.

Frequently occurring rhythms contain many data samples, while relatively rare T and SVTA contain fewer data samples. These result in data imbalance; thus, when configuring the data of a mini-batch in the learning process, different sampling weights were designated for each rhythm. Each mini-batch was obtained by weighted random sampling, and the value of the batch size was 16.

### 4.2. Hyperparameters and Settings

CNN weights were learned by transfer learning; hence, after a pre-trained initialized weight was called, the learning was carried out using an Adam optimizer. To search for the optimal values for hyperparameters, such as the learning rate and the number of epochs, a grid search was performed on the validation dataset. The learning rate was chosen as the best value in the range of 0.1 to 0.0001, and the number of epochs was chosen as the best value in the range of 10 to 50. As a result, the optimal performance on the validation dataset was at a learning rate of 0.001 and an epoch of 30. We also set the batch size to 4, which is the largest value that can be selected in the experimental environment settings. Five-fold cross-validation was conducted on all experiments.

## 5. Results

N and AFIB are studied often; thus, they were included. Two ECG rhythm datasets were created, and an experiment was conducted on these datasets. The first dataset consisted of rhythms with fewer samples than N and AFIB. Through this, we evaluated the ability of the proposed algorithm to distinguish N and AFIB simultaneously and differentiate rhythm classes with relatively few samples. The second rhythm dataset, which was created to assess the ability of the proposed algorithm to distinguish between AFIB and AFL, is discussed as a limitation of existing studies. For this goal, AFIB, AFL, N, and three additional ECG rhythms were included. We analyzed the ability of the proposed model for this rhythm dataset to classify AFIB and AFL.

The performance of the proposed algorithm was analyzed with respect to its accuracy (Acc), precision (Pre, known as PPV), sensitivity (Sen, known as recall), specificity (Spec), and F1 score.

### 5.1. Experiment to Classify ECG Rhythm with Few Samples

First, an experiment was conducted on the first rhythm dataset, and the five rhythm classes used were N, AFIB, SVTA, B, and T. The number of data samples for each rhythm is shown in Table 1. The results of the experiment are summarized in Table 3. The overall Acc was 99.08%, and the proposed method is suitable for rhythm classes with a relatively small number of data samples (e.g., B, T, and SVTA). Notably, the model was able to distinguish SVTA with the smallest number of samples with 100% accuracy.

A confusion matrix verifying the classification performance for each class is presented in Table 4. According to the matrix, T is often misclassified as N because the definition of T is the ECG signal that generates a prematurity ventricular contraction after two normal beats in this class. Thus, a significant number of normal beats are included in the sequence, which makes it difficult for the model to differentiate between T and N.

### 5.2. Experiment for ECG Rhythms That Are Difficult to Distinguishable

The following is an experiment evaluating the performance of the proposed method using the second rhythm dataset. A total of 6 six rhythms, N, AFIB, P, SBR, B, and AFL, were tested. The number of samples for each rhythm is summarized in Table 2.

**Figure 4:**
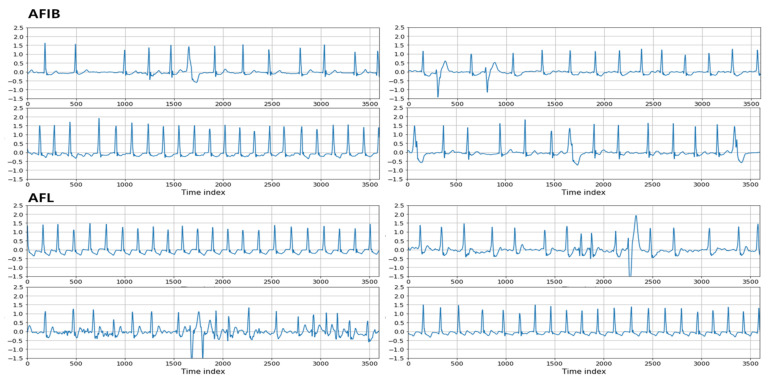
Examples of AFIB and AFL rhythms.

The purposes of this experiment include evaluating the ability of the proposed method to classify different ECG rhythms, including AFIB and AFL, with similar characteristics and determining whether the proposed method can be supplemented well. First, the appearance of the ECG waveforms of AFIB and AFL was confirmed in Figure 4. After comparing AFIB and AFL, we found that some parts are different, while many parts are similar. Thus, we tried to distinguish AFIB and AFL, which are hard to separate, by distinguishing various ECG rhythms with the proposed algorithm.

The results of this experiment are summarized in Table 5, which demonstrates that the proposed method was able to classify all the considered rhythms. The overall accuracy was 99.24%, while the F-1 score was ~99%, except for the AFL. Based on the confusion matrix shown in Table 6, we found that the proposed algorithm was successful in classifying AFIB and AFL. Therefore, all rhythms except AFL are well classified. In the case of AFL, ~9% of AFL rhythms were misclassified as AFIB. Nevertheless, the proposed method shows significant performance for AFIB and AFL.

### 5.3. Comparison with a Recent Study

To compare the performance of the proposed method with existing methods, we refer to a recent paper that studied the same data and rhythms. The previous paper, for comparison with the proposed method in the first rhythm dataset, implemented a rhythm classification that combines beat unit and spectrogram unit features of an ECG signal [27]. A comparison of the proposed and previous models relative to small ECG rhythm samples is shown in Table 7. The F1-score was used to evaluate their performance. The proposed method showed better overall performance, and the performance for SVTA rhythms has improved particularly by >20%. The poor performance of the previous method is due to overfitting in N and AFIB, whereas our method classified the rhythms without overfitting due to the presence of many samples.

To evaluate the performance of the second rhythm dataset, a previous paper proposed a method merging the 1D signal and R–R interval as inputs of CNN using an ECG signal in one dimension and network, classifying rhythms through this approach [26]. A comparison of the performance obtained for the previous and proposed methods on each class using the F1-score is shown in Table 8. The performance of the proposed method for classifying AFIB and AFL improved by about 37% compared with the previous study. Additionally, the performance of other rhythms is ameliorated by 2%–3%.

To investigate the effect of noise in ECG signals on the performance of the proposed method, we performed additional experiments. To this end, some randomly generated noise was added to ECG signals at two different signal-to-noise ratio (SNR) levels of 6 dB and −6 dB, respectively. These noise-added ECG signals were used to produce BSM images and train a beat classification model. The F1-score results of rhythm classification with noise ECG signals are shown in Table 9. Some noises up to the SNR level of 6 dB do not appear to affect the rhythm classification performance of our proposed method. On the other hand, as the SNR level increases to −6 dB, the overall performance degrades significantly. The overall accuracy also dropped from 99.24% to 94.03% at an SNR level of −6 dB.

## 6. Conclusions and Discussion

We classified various ECG rhythms through the proposed algorithm. We solved the problems caused by differences in the number of data samples and distinguished between AFIB and AFL, which have similar characteristics. Our method converts ECG signals into a new type of image, which we refer to as a BSM image, and it classifies the created image through CNN. The BSM image was designed to consider previously used features, such as the R–R interval and the listing pattern of ECG beats. The proposed method can be used in classifying difficult rhythms with few samples. It can also be used to distinguish AFIB and AFL with similar characteristics. Based on this, it seems that the method can be helpful in distinguishing rhythms that are less frequent but dangerous and similar but different.

There are some limitations of this study that need to be explored in future work. For example, the locations of the R peaks obtained from the database were used to create the BSM image; hence, when using databases that do not have information about the locations of R peaks, peak detection is required separately. If there are many incorrect detection results, the BSM image will be affected; thus, the overall performance of the method may decrease. 

## Figures and Tables

**Table 1:** Number of samples in the 5-class dataset used to classify ECG rhythms with few samples. Abbreviations are defined in the text.

5-Class	N	AFIB	B	T	SVTA
Number of samples	8756	7215	823	410	91

**Table 2:** Number of samples in the 6-class dataset difficult to distinguish ECG rhythms with similar characteristics, such as AFIB and AFL. Abbreviations are defined in the text.

6-Class	N	AFIB	P	SBR	B	AFL
Number of samples	8756	7215	1796	1796	823	549

**Table 3:** Performance of classification model for ECG rhythms with a small number of samples.

Class	Spec (%)	Sen (%)	Pre (%)	F1 Score (%)	Acc (%)
N	99.45	98.72	99.52	99.12	99.11
AFIB	98.74	99.56	98.98	99.27	99.39
SVTA	99.08	100	100	100	100
B	99.05	99.76	98.92	99.33	99.94
T	99.14	96.83	92.11	94.41	99.73

**Table 4:** Confusion matrix of the proposed approach for ECG rhythms with few samples.

	N	AFIB	SVTA	B	T
N	8644	70	0	8	34
AFIB	31	7183	0	1	0
SVTA	0	0	91	0	0
B	1	1	0	821	0
T	10	3	0	0	397

**Table 5:** Classification performance for ECG rhythms with similar characteristics.

Class	Spec (%)	Sen (%)	Pre (%)	F1 Score (%)	Acc (%)
N	99.10	99.44	99.76	99.60	99.67
AFIB	99.27	99.18	98.72	98.95	99.27
B	99.21	99.88	99.04	99.46	99.96
P	99.17	100	100	100	100
AFL	99.47	90.84	93.06	91.94	99.58
SBR	99.17	100	100	100	100

**Table 6:** Confusion matrix for classification of indistinguishable ECG rhythms.

	N	AFIB	B	P	AFL	SBR
N	8707	43	6	0	0	0
AFIB	20	7156	2	0	37	0
B	1	0	822	0	0	0
P	0	0	0	1796	0	0
AFL	0	50	0	0	496	0
SBR	0	0	0	0	0	1796

**Table 7:** Comparison of proposed and recent (Pokaprakarn et al., 2021) methods for small ECG rhythm samples.

Model	N	AFIB	B	T	SVTA
Proposed method	99.12	99.27	**100**	**99.33**	**94.41**
Pokaprakarn [27]	98.62	95.79	78.35	84.22	87.22

**Table 8:** Comparison of proposed and recent (Chen et al., 2020) methods for rhythms with similar characteristics.

Model	N	AFIB	B	P	AFL	SBR
Proposed method	99.60	98.95	99.46	100	**91.94**	100
Chen [27]	99.64	96.26	96.43	99.68	54.55	98.35

**Table 9:** Effect of noise in ECG signals on the performance of the proposed method.

SNR Level	N	AFIB	B	P	AFL	SBR
Original data	99.60	98.95	99.46	100	91.94	100
6 dB	98.58	96.32	97.83	100	70.73	100
−6 dB	96.03	93.08	95.00	94.97	57.48	99.17

## Data Availability

All data used in the experiment are provided by Physionet and are available at the following address: https://physionet.org/content/mitdb/1.0.0/ (accessed on 3 January 2023).
